# Navigating Concurrent Diagnoses With Cancer and Mental Health Disorder: Patients' Perspectives on Personalised Treatment and Care

**DOI:** 10.1111/jocn.70367

**Published:** 2026-05-21

**Authors:** Kim Jørgensen, Maria Estrup Scredi, Kathrine Holmstrøm Ougaard, Hanne Elisabeth Bagger Hansen, Kimmie Juul Heine, Sofie Schuster, Helle Pappot, Karin Piil

**Affiliations:** ^1^ Department of Health Services Research, User Perspectives and Community‐Based Initiatives, Faculty of Health Sciences University of Southern Denmark (SDU) Odense Denmark; ^2^ Department of People and Technology Roskilde University Roskilde Denmark; ^3^ Department of Otorhinolaryngology, Head and Neck Surgery, and Audiology Copenhagen University Hospital, Rigshospitalet Copenhagen Denmark; ^4^ University College Absalon Sorø Denmark; ^5^ Rigshospitalet Copenhagen University Hospital Copenhagen Denmark; ^6^ Aarhus University Aarhus Denmark; ^7^ Centre for Health, Prevention and Elder Care Frederikssund Denmark; ^8^ Department of Oncology, Centre for Cancer and Organ Diseases Copenhagen University Hospital, Rigshospitalet Copenhagen Denmark; ^9^ Department of Clinical Medicine, Faculty of Health and Medical Sciences University of Copenhagen Copenhagen Denmark; ^10^ School of Nursing, Faculty of Health Sciences Curtin University Perth Western Australia Australia

**Keywords:** cancer care, continuity of care, co‐morbidity, diagnostic overshadowing, mental health conditions, oncology nursing, patient experience, patient‐centred care, psychosocial oncology, qualitative research

## Abstract

**Background:**

People living with concurrent cancer and mental health disorders face heightened vulnerability within healthcare systems that are predominantly organised around biomedical treatment pathways. Although cancer care has advanced substantially, less is known about how these patients experience recognition of their psychological vulnerability, continuity of care, communication and opportunities to participate meaningfully in decisions about treatment.

**Aim:**

To explore patients' experiences of living with concurrent cancer and mental health disorders, with particular focus on how they experience being met in their individual needs within oncology care.

**Methods:**

This qualitative study used individual semi‐structured interviews with 11 adult patients receiving current or recent cancer treatment and living with an existing mental health or neurodevelopmental condition. Participants were recruited purposively from oncology and haematology departments at two Danish hospitals between September 2024 and December 2025. Data were analysed using interpretive qualitative content analysis.

**Results:**

Six interrelated themes were identified: (1) being treated for cancer while mental illness was left outside the room; (2) the hidden work of self‐coordination in a fragmented system; (3) communication as care: the need for predictability and adaptation; (4) cancer treatment as a trigger for mental health deterioration; (5) stigma, legitimacy and self‐silencing; and (6) relational continuity and being remembered as protective factors. Across themes, participants described a persistent tension between technically competent somatic treatment and insufficient recognition of psychological vulnerability, support needs and relational needs.

**Conclusion:**

Patient‐centred oncology care for people living with co‐morbidity depends not only on clinical expertise, but on recognition, adapted communication, shared responsibility and relational continuity. The findings suggest that vulnerability is shaped not only by illness itself, but also by how care systems and professional practices are organised.

**Relevance to Clinical Practice:**

Small but meaningful changes in everyday oncology practice including psychologically informed communication, continuity of contact persons, explicit recognition of mental health needs and shared coordination of care—may substantially improve safety, participation and patient experience for this population.

**Patient or Public Contribution:**

Patients living with concurrent cancer and mental health disorders contributed to this study through individual qualitative interviews. Their lived experiences formed the empirical foundation of the study and informed the analysis and interpretation of findings. Patients were not involved in the design of the study or the preparation of the manuscript.

## What Does This Paper Contribute to the Wider Global Clinical Community?

- Patients living with concurrent cancer and mental health disorders experience oncology care as highly competent yet insufficiently responsive to psychological vulnerability and relational needs.
- Patient‐centred care for this population relies on recognition, adapted communication, relational continuity and shared responsibility rather than additional specialist services alone.
- The study provides empirical insight into how fragmented care structures and assumptions about patient autonomy may unintentionally increase vulnerability among patients with complex mental health needs.

## 1 Introduction

People living with concurrent cancer and mental health disorders are placed in a particularly complex and vulnerable position within healthcare systems. Individuals with co‐morbidity must navigate not only the physical demands and side effects of cancer treatment but also the emotional, cognitive, and psychological challenges associated with mental illness. The interaction between these conditions often intensifies symptom burden and complicates patients' ability to engage in, tolerate, and adhere to cancer treatment (Brook and Amaro Calcia 2016; Jasmin et al. 2019). Cancer treatments such as chemotherapy and radiation therapy are frequently associated with severe side effects, including fatigue, pain, nausea and immunosuppression. For patients with pre‐existing or concurrent mental health disorders such as depression, anxiety, or mood disorders these physical stressors may exacerbate psychological distress and undermine coping capacity (Shalata et al. 2024). Research shows that cancer‐related distress is closely linked to increased symptoms of anxiety and depression, reduced quality of life, and difficulties in maintaining everyday functioning (Papadopoulou et al. 2022; Ramathuba and Ramutumbu 2025; Weber and O'Brien 2017). For patients with co‐morbidity, these challenges are often further intensified by stigma related to mental illness. Despite growing awareness, negative attitudes and misconceptions persist within healthcare contexts, potentially influencing how patients are perceived, listened to and supported. Patients may experience that their psychological symptoms are overlooked, minimised, or interpreted as non‐compliance, which can undermine trust and discourage help‐seeking (Bergerot et al. 2024; Grassi et al. 2025; Vass et al. 2025). Stigma may also shape clinical encounters by constraining communication and limiting opportunities for patients to articulate their needs and concerns (Phelan et al. 2023). From a patient perspective, managing cancer alongside mental illness often involves navigating fragmented care pathways, competing treatment priorities, and involvement of multiple healthcare professionals and services with differing responsibilities, priorities and treatment perspectives. Patients may experience difficulties in having their needs recognised as a whole, rather than as separate physical and psychological conditions. The burden of coordinating appointments, understanding treatment plans and making complex decisions can become overwhelming, particularly when cognitive or emotional symptoms are present (Aoki et al. 2022; Rayner et al. 2023). A patient‐centred approach is therefore essential when caring for individuals with co‐morbidity. Understanding patients lived experiences provides critical insight into how care is perceived, where gaps occur and how healthcare encounters can either support or hinder patients' sense of agency and dignity. Incorporating patients' perspectives into care planning has been shown to improve psychological well‐being, enhance treatment adherence and foster empowerment by aligning care with patients' values, preferences and life situations (Mertens et al. 2025; Punnett et al. 2024; Shickh et al. 2023). Mental health conditions are common among people living with cancer and represent a substantial clinical and organisational challenge internationally (Grassi et al. 2023, 2025). Depression, anxiety, trauma‐related symptoms and severe pre‐existing mental health disorders may occur before, during, or after cancer diagnosis and treatment (Papadopoulou et al. 2022; Shalata et al. 2024). These co‐occurring conditions are associated with delayed help‐seeking, lower participation in screening programmes, reduced access to timely treatment, poorer treatment adherence, increased use of acute services and greater psychosocial burden for patients and families (Bergerot et al. 2024; Schäfer et al. 2026; Vass et al. 2025).

People living with mental health disorders often face substantial inequities across the cancer pathway, from symptom recognition and help‐seeking to diagnosis, treatment and survivorship. International studies have shown that stigma, diagnostic overshadowing and structural barriers may contribute to delayed presentation, reduced participation in screening programmes and lower access to timely cancer investigation and treatment (Grassi et al. 2023; Vass et al. 2025). Mental health symptoms such as anxiety, depression, cognitive difficulties, or social withdrawal may further complicate recognition of bodily symptoms and engagement with healthcare services (Bergerot et al. 2024; Myers et al. 2025). Previous studies have also reported poorer continuity of care and difficulties participating in shared decision‐making among patients with mental health disorders within somatic healthcare settings (Kim et al. 2024; Mertens et al. 2025). Despite growing international attention to these disparities, less is known about how patients themselves experience being met within everyday oncology practice when living with concurrent cancer and mental health disorders. In particular, qualitative insight is needed into how communication, continuity, recognition of vulnerability and organisational care structures shape patients' sense of safety, dignity and ability to engage in treatment. Such knowledge is important for developing more integrated, responsive and ethically grounded models of cancer care (Coulter and Jenkinson 2005; Granek et al. 2019b; Merrick et al. 2022).

In this study, patient‐centred care is understood as care that responds to patients' values, preferences, capacities and life circumstances rather than focusing solely on disease management. Agency refers to patients' ability to participate meaningfully in decisions and actions concerning their care, while recognition refers to being acknowledged as a whole person whose psychological, relational and somatic needs are all clinically relevant. These concepts provide an interpretive lens for exploring how patients experience oncology care when living with concurrent cancer and mental health conditions.

### 1.1 Aim

The aim of this study was to explore patients' experiences of living with concurrent cancer and mental health disorders, with particular focus on how they experience being met in their individual needs within oncology care and how this influences their sense of agency, well‐being and participation in care.

## 2 Methods

### 2.1 Design

This study was positioned within an interpretive qualitative research tradition, aiming to explore how participants make sense of their experiences within specific contexts (Polit and Beck 2021; Thorne 2025). An interpretive approach was considered appropriate because the aim was to explore how patients made meaning of their experiences of oncology care while living with concurrent cancer and mental health conditions. Qualitative content analysis was chosen to enable systematic interpretation of both manifest and latent meanings within participants' accounts and to foreground participants' lived experiences within a clinical context.

### 2.2 Study Setting and Sampling

Participants were recruited from the Departments of Oncology and Haematology at a large hospital in Copenhagen and from the Department of Oncology at Zealand University Hospital between September 2024 and December 2025. Purposive sampling was used to seek variation in cancer diagnoses, treatment trajectories and types of mental health disorders. Although purposive sampling may introduce selection bias (Ames et al. 2019), this approach was considered appropriate for generating rich, experience‐based accounts relevant to the study aim. Patients were informed about the study by healthcare professionals or through written patient information materials and contacted the research team directly if interested. As recruitment occurred within clinical settings, the possibility of gatekeeping cannot be fully excluded. Healthcare professionals may have influenced which patients received study information based on considerations such as treatment burden, emotional vulnerability, or perceived suitability for participation.

Participation was entirely voluntary, and patients who expressed interest and met the inclusion criteria were invited to participate. During the recruitment period, purposive sampling was used to monitor variation in age, gender, cancer diagnosis, treatment trajectory and type of mental health disorder. No eligible patients who contacted the research team were excluded on the basis of gender or diagnosis alone. Inclusion criteria were: (1) adults aged 18 years or older, (2) current or recent cancer treatment and (3) an existing clinically recognised mental health or neurodevelopmental condition associated with psychological vulnerability or support needs. Diagnoses were based on participants' existing medical records or previously established clinical diagnoses within routine care and were not independently assessed by the research team for the purpose of this study. Exclusion criteria were inability to provide informed consent, severe physical or psychological distress considered to make participation inappropriate at the time of recruitment, or inability to participate in an interview conducted in Danish. Efforts were made to ensure diversity in age, gender, diagnosis and treatment phase in order to capture a broad range of experiences (Table 1). The participant group included a heterogeneous range of conditions, including mood disorders, anxiety disorders, personality disorders, neurodevelopmental conditions and other complex mental health presentations. Diagnostic labels are presented descriptively and reflect existing clinical documentation.

**TABLE 1:** Participants.

Participant	Gender	Age	Cancer diagnosis	Mental illness	Years with cancer	Years with mental illness
1	Female	59	Breast cancer with lymph node involvement	Chronic suicidality	< 1 year	Many years
2	Female	55	Breast cancer (cancer‐free)	Autism spectrum disorder (Asperger's)	Approx. 1 year	Lifelong
3	Female	43	Ovarian cancer with metastases	ADHD, paranoid schizophrenia	< 1 year	Many years
4	Female	57	Ovarian cancer	Asperger's syndrome, depression	< 1 year	Many years
5	Male	62	Oesophageal cancer with metastases	Anxious–avoidant personality disorder, anxiety/depression	< 1 year (recurrence)	> 30 years
6	Female	52	Breast cancer	Anxiety, ADHD, emotionally unstable personality disorder	< 1 year	> 5 years
7	Female	49	Breast cancer	Bipolar disorder	< 1 year	Approx. 8 years
8	Female	50	AML (acute leukaemia)	PTSD	< 1 year	Approx. 6–7 years
9	Female	46	Ovarian cancer (stage II–IV)	Panic disorder, OCD, ADHD	< 1 year	> 25 years
10	Female	47	Cervical cancer	ADD, anxiety, avoidant personality traits	< 1 year	Many years
11	Female	55	Breast cancer (2023) + secondary AML; currently in follow‐up after stem cell transplantation	Personality disorder; depressive symptomatology	Approx. 2 years	Many years

### 2.3 Data Collection

Individual semi‐structured interviews were conducted with all participants. Interviews lasted approximately 30–60 min and took place either in a private room at the hospital or online via Teams or telephone, depending on participant preference. The interview guide was developed to explore patients' experiences of living with concurrent cancer and mental health disorders and their encounters with oncology care. Its development was informed by the study aim, existing literature on patient‐centred care, psychosocial vulnerability in cancer care, stigma, continuity of care and communication needs among patients with complex health conditions. In addition, the guide was inspired by qualitative interviewing principles described by (Brinkmann and Kvale 2007) and by (McCance and McCormack 2025) emphasising open‐ended, exploratory and experience‐near questions. The guide included questions and prompts related to experiences of diagnosis and treatment, communication with healthcare professionals, involvement in decisions, coordination across services, emotional support and perceptions of being recognised and met according to individual needs. The interview guide was used flexibly, allowing participants to introduce issues of personal importance and enabling follow‐up questions to deepen emerging themes (Table 2).

**TABLE 2:** Interview guide.

Interview domain	Exploratory focus	Example questions
Personal background	Current life situation	Can you tell me about yourself and your everyday life?
Living with both conditions	Everyday challenges	How has cancer affected your mental health?
Experiences with oncology care	Encounters with professionals	How do you experience being met in oncology care?
Communication and information	Understanding and involvement	How is information communicated to you?
Coordination of care	Pathways across services	How do you experience coordination between services?
Mental health during treatment	Changes and support needs	Have you experienced changes in your mental health during treatment?
Talking about distress	Openness and barriers	Have you hesitated to talk about your mental health?
Support systems	Helpful people/resources	Who has been most supportive?
Closing reflections	Additional perspectives	What should healthcare professionals better understand?

The sample size was guided by considerations of information power, whereby participants were recruited until the data were judged to provide sufficient richness, relevance and variation to address the study aim. During the later interviews, recurring patterns relating to communication, stigma, fragmented care and relational continuity suggested that the dataset was sufficiently robust to support the analysis. The relationship between researchers and participants was carefully considered throughout the study. Interviews were conducted by experienced qualitative researchers with professional backgrounds in health research and an interest in patient experiences of complex care pathways. No interviewer had a direct clinical treatment relationship with participants. Participants were informed about the purpose of the study, the voluntary nature of participation and the researchers' independent role prior to the interviews. Given the sensitivity of discussing cancer and mental health experiences, attention was paid to creating a respectful, safe and non‐judgemental interview environment. Interviewers sought to facilitate open dialogue through active listening, empathic communication and flexible pacing of interviews according to participants' needs. Reflexivity was maintained throughout the research process. The researchers continuously reflected on how their professional backgrounds, assumptions and prior knowledge of healthcare systems might influence data generation, interpretation and theme development. Analytical decisions and emerging interpretations were therefore discussed within the author group to challenge preconceptions and strengthen credibility. The researchers also acknowledged that their professional backgrounds and commitment to patient‐centred care may have sensitised them to issues of vulnerability, communication and coordination. Reflexive dialogue was therefore used to remain open to alternative interpretations.

Throughout the analytical process, the researchers remained attentive to how their professional backgrounds and prior understandings of healthcare systems might shape interpretation. Reflexive dialogue within the author group was used to challenge assumptions and consider alternative readings of the data.

### 2.4 Data Analysis

Interviews were audio‐recorded, transcribed verbatim and analysed using qualitative content analysis as described by (Graneheim and Lundman 2004; Lyhne et al. 2025). NVivo software was used to support data management and transparency. Analysis was conducted collaboratively by all authors through an iterative process. Differences in interpretation were addressed through discussion and repeated return to the original transcripts until consensus was reached. Divergent readings were treated as analytically valuable and used to refine categories and themes. Transcripts were read repeatedly to gain an overall understanding, followed by identification and condensation of meaning units related to the research aim. Codes were developed and discussed within the research team, then grouped into sub‐categories and categories. The analysis moved iteratively between manifest content (participants' explicit descriptions and experiences) and latent content (underlying meanings, assumptions and patterns across accounts). Finally, overarching themes were constructed to capture latent meanings across the data, focusing on how patients experience being met or not met in their individual needs within oncology care. The seven analytical steps are outlined in Table 3. Theme development was iterative and interpretive. Preliminary codes and categories were compared across interviews to identify recurring patterns as well as meaningful variation in participants' experiences. Candidate themes were then discussed collaboratively within the author group and refined through repeated return to the original transcripts. Themes were reviewed against the full dataset to assess internal coherence, distinction between themes and consistency with participants' accounts before final naming. Final theme titles were selected to capture the latent meaning across several related categories while remaining grounded in participants' own accounts and language. Several strategies were used to enhance credibility and trustworthiness, including collaborative analysis across authors, maintenance of an audit trail of coding and theme development, reflexive discussions throughout the analytic process and continuous comparison between emerging interpretations and the original transcripts. Formal member checking was not undertaken, as the study prioritised interpretive depth rather than participant validation of thematic constructions. A completed COREQ 32‐item checklist is provided as Supporting Information S1.

**TABLE 3:** The content analysis.

Step in analysis	Analytical process	Example from interview transcript	Output of the step
1. Meaning unit	Identification of text segments relevant to the aim	‘It is a double burden to have both mental vulnerability and cancer… in the somatic system, the psychological part is forgotten.’ (Woman, Participant 6)	Experience of psychological needs being overlooked in cancer care
2. Condensation	Shortening the meaning unit while preserving core meaning	Psychological vulnerability is overlooked in somatic cancer care	Essential meaning retained
3. Code	Labelling the condensed meaning	Mental illness left outside oncology care	Code captures manifest content
4. Sub‐category	Grouping similar codes	Codes such as: *mental health not addressed*, *distress seen as normal reaction*, *psychological needs ignored*	Sub‐category: Psychological vulnerability marginalised
5. Category	Abstracting sub‐categories into broader concepts	Psychological vulnerability marginalised + somatic focus dominates care	Category: Hierarchies and fragmentation in care
6. Theme	Interpreting latent meaning across categories	Hierarchies and fragmentation reflect how systems prioritise cancer while sidelining mental illness	Theme: Being treated for cancer while mental illness is left outside the room

### 2.5 Ethical Considerations

The study was conducted in accordance with the Declaration of Helsinki World Medical Association (2013) and relevant national legislation governing health research and data protection (Ministry of the Interior and Health 2020). Ethical approval was granted by the Regional Committees for Medical and Health Research Ethics (REK Norway), reference no. 777200, on 11 September 2024. In Denmark, the project has been registered and approved by the Research Legal Department of the Capital Region of Denmark (project ID: p‐2024‐17450) and complies with the Danish Data Protection Act. Managerial approval was obtained from the participating oncology departments at Rigshospitalet, Copenhagen University Hospital and Zealand University Hospital. As the study does not involve any physical or psychological intervention, formal approval from a biomedical research ethics committee in Denmark was not required. All participants received written and oral information about the study's purpose, procedures, potential risks and benefits prior to participation. Written informed consent was obtained from all participants. Participation was voluntary, and participants were explicitly informed of their right to withdraw from the study at any time and without consequences for their ongoing or future care. Participants were also informed that, should they choose to withdraw, their interview data could be deleted upon request. Given the vulnerability of the study population, particular attention was paid to safeguarding participants' well‐being during the interview process. Interviews were conducted with sensitivity, and participants were free to pause or terminate the interview if they experienced discomfort or distress. At the conclusion of each interview, participants were offered time to debrief, ask questions and reflect on their experience of participation. Particular attention was given to participants' emotional well‐being following discussions of potentially sensitive topics related to cancer and mental health. Where relevant, participants were encouraged to contact their usual healthcare professionals or established support services if distress arose after the interview. Participants were also provided with contact details for the research team should they have further questions or concerns following participation.

All data were handled confidentially and stored securely on encrypted servers located within the hospital regions' protected IT infrastructure. Access to data was restricted to authorised members of the research team only. Audio files and transcripts were anonymised prior to analysis to prevent identification of individual participants. In accordance with data protection regulations, all research data will be stored securely and permanently deleted in 2030.

## 3 Findings

The qualitative content analysis of the 11 patient interviews identified six interrelated themes that describe how patients living with concurrent cancer and mental health disorders experience being met in oncology care. Across interviews, patients described a persistent tension between highly competent biomedical cancer treatment and insufficient recognition of psychological vulnerability. The themes reflect shared patterns across the dataset rather than individual trajectories and illuminate how organisational structures, professional practices and relational encounters shape patients' experiences of safety, agency and dignity. At the same time, participants' experiences varied in intensity, available support and capacity to navigate care, revealing important differences as well as common patterns. While presented separately for analytic clarity, the themes were closely interconnected in practice.

### 3.1 Theme 1. Being Treated for Cancer While the Mental Illness Is Left Outside the Room

Across interviews, patients consistently described oncology care as efficient, well organised and focused on cancer as a somatic disease. Although mental health diagnoses were often documented, patients rarely experienced that this knowledge informed how care was delivered. Psychological distress was frequently treated as an expected reaction to cancer rather than as part of a recognised mental health condition.

Patients described feeling only partially seen, acknowledged as cancer patients but not as individuals living with intersecting vulnerabilities.

It is a double burden to have both mental vulnerability and cancer… in the somatic system, the psychological part is forgotten. (Woman, Participant 6)

Because I am mentally ill, it becomes so difficult to manage everything. (Woman, Participant 1)

At a latent level, this theme reflects a hierarchy of illness within healthcare, where somatic disease is prioritised as legitimate and urgent, while mental illness is marginalised. Patients experienced this separation as emotionally isolating, particularly during periods of heightened vulnerability.

### 3.2 Theme 2. The Hidden Work of Being One's Own Coordinator in a Fragmented System

A dominant theme was patients' experience of becoming responsible for coordinating care across oncology, psychiatry, primary care and social services. This work was experienced as exhausting and overwhelming, especially during treatment phases marked by fatigue and psychological distress.

Patients described how they were expected to manage appointments, transport and information flow between sectors without support

It means that I have to coordinate everything myself and it is overwhelming. (Woman, Participant 6)

I end up acting as a kind of intermediary, and that is hard when you are already ill. (Woman, Participant 7)

This theme reveals a structural mismatch between healthcare systems organised around assumptions of patient autonomy and the lived realities of patients with mental health disorders. Responsibility was shifted onto patients at times when their capacity was most limited. This theme points not only to organisational fragmentation, but also to implicit assumptions that patients possess the cognitive, emotional and practical capacity to coordinate complex care across sectors.

### 3.3 Theme 3. Communication as Care: The Need for Predictability and Adaptation

Patients consistently linked feeling safe and involved in their care to how information was communicated. Standardised, fast‐paced consultations and large amounts of verbal information were often overwhelming, particularly for patients with anxiety, attention difficulties, or cognitive overload.

Several patients described difficulties remembering information when feeling pressured or distressed. While feeling overwhelmed during cancer consultations may not be unique to this group, participants described how pre‐existing anxiety, attention difficulties, cognitive overload, or trauma‐related stress responses intensified these challenges. For several participants, standardised and information‐dense consultations became particularly difficult to manage, increasing the need for repetition, written information, predictability and emotional reassurance.

It wasn't written down… I can't remember it when I feel under pressure. (Woman, Participant 2)

I bring a small notebook with questions to the consultations. (Woman, Participant 6)

At a deeper level, this theme highlights that communication is not merely informational but a core element of care. When communication is poorly adapted, patients' ability to participate and make informed decisions is undermined. Predictability, repetition and written information supported agency and reduced anxiety. Communication difficulties were therefore not solely interpersonal, but also shaped by time pressure, standardised consultation formats and limited adaptation to individual vulnerability.

### 3.4 Theme 4. When Cancer Treatment Triggers Mental Health Deterioration

Across interviews, patients described how cancer treatment intensified existing mental health symptoms, including anxiety, panic, insomnia and mood instability. Despite this, few experienced that such effects were anticipated or systematically addressed within oncology care.

Patients emphasised that mental health deterioration was often experienced as sudden and destabilising.

I became completely sped up and couldn't sleep… it would have helped if they had taken more account of the fact that I have bipolar disorder. (Woman, Participant 7)

Panic anxiety and fear of death… cancer has made it much worse. (Woman, Participant 9)

This theme reflects a lack of anticipatory and integrated care. When mental health symptoms escalated, patients often felt left to manage on their own, increasing vulnerability during treatment.

### 3.5 Theme 5. Stigma, Legitimacy and the Risk of Withholding Distress

A recurring undercurrent across interviews was patients' concern about not being taken seriously due to their mental health diagnoses. Some described hesitating to disclose distress or minimising symptoms to avoid being perceived as difficult or unstable.

Patients described feeling unsure whether their experiences would be considered legitimate.

It can be difficult when you have a mental health diagnosis, because you may feel that you are not taken seriously. (Woman, Participant 7)

I have tried to get the oncologists to take my mental health into account, but it doesn't seem to be part of their practice. (Woman, Participant 6)

This theme illustrates how stigma operates subtly through silence, non‐response, or lack of inquiry rather than overt discrimination. Fear of delegitimisation contributed to self‐censorship, potentially compromising emotional support and clinical safety.

### 3.6 Theme 6. What Helps: Relational Continuity and Being Remembered

Despite systemic challenges, patients described forms of support that made a meaningful difference. These supports were primarily relational rather than organisational and included continuity of care, trusted companions and professionals who showed genuine interest and presence.

Patients emphasised the importance of not facing illness alone

I have a sister who often comes with me. She is my anchor. (Woman, Participant 6)

I am amazed at how welcoming and kind people can be. (Man, Participant 5)

Feeling recognised as a person rather than a diagnosis restored a sense of dignity and belonging. Such relational anchors mitigated fragmentation and helped patients navigate care despite systemic shortcomings. For some participants, continuity was created through specific professionals, while for others it depended on relatives or personal coping strategies, illustrating variation in how support was accessed.

Across themes, fragmented organisation, insufficient recognition of mental health needs and poorly adapted communication often reinforced one another. Conversely, when patients experienced relational continuity, recognition and shared responsibility, their sense of safety and ability to participate in care increased. The findings therefore suggest that vulnerability was shaped not only by illness itself, but by the way care structures and relationships were organised.

## 4 Discussion

This study explored how patients living with concurrent cancer and mental health disorders experience being met in oncology care. Across 11 patient interviews, six interrelated themes illuminated a persistent tension between highly competent biomedical cancer treatment and insufficient recognition of psychological vulnerability. In this discussion, we synthesise the findings across themes, situate them within existing research and articulate the novel contributions of the study. Overall, the findings demonstrate that being ‘met according to individual needs’ is not primarily about supportive care or specialised interventions, but about recognition, relational continuity and shared responsibility within care systems that are otherwise highly standardised and somatically oriented.

In this study, patient‐centred care is understood not merely as responsiveness to preferences, but as the practical organisation of care around patients' capacities, vulnerabilities and relational needs. Agency is conceptualised as relational and conditional rather than purely individual, emerging when patients are supported to understand, participate and act within care processes. Recognition refers to patients being acknowledged as whole persons whose psychological, social and somatic needs are all clinically relevant. Viewed through this lens, the findings suggest that deficits in care were not only interpersonal, but organisational and structural.

### 4.1 Fragmentation of Care and the Hierarchy of Illness

A central contribution of this study is the empirical demonstration of how patients experience a hierarchy of illness in oncology care, where cancer is treated as the legitimate and urgent condition, while mental illness is marginalised or externalised. Although mental health diagnoses were often documented, patients rarely experienced that this knowledge shaped communication, follow‐up, or treatment planning. This finding extends previous research showing that mental health needs in oncology are frequently under‐recognised and under‐addressed (Bergerot et al. 2024; Grassi et al. 2025) by illustrating how this marginalisation is experienced from the patient perspective as *partial recognition* being treated as a cancer patient, while their mental health needs receive less attention.

This hierarchy has important consequences. When psychological distress is normalised as an ‘expected reaction’ to cancer, it paradoxically becomes invisible as a clinical concern. Similar patterns have been described in psychosocial oncology, where distress screening exists but is not consistently translated into action (Papadopoulou et al. 2022; Weber and O'Brien 2017). Our findings add nuance by showing that for patients with pre‐existing mental health disorders, this invisibility is not harmless; it amplifies vulnerability, isolation and the sense of being left alone with the psychological consequences of cancer. The Burden of Coordination and the Limits of Patient Autonomy.

Across themes, patients described being positioned as the primary coordinators of their own care across oncology, psychiatry, primary care and social services. This ‘hidden work’ of coordination emerged as a major source of distress, particularly during periods of fatigue, anxiety, or cognitive overload. While patient autonomy is often framed as empowering, our findings demonstrate how autonomy can become burdensome when it is assumed rather than supported.

This insight contributes to existing literature on fragmented care pathways for people with complex needs (Aoki et al. 2022; Rayner et al. 2023). While previous studies have highlighted system‐level fragmentation, our study shows how fragmentation is *embodied* by patients, who must manage transitions, information flow and responsibility at times when their capacity is most compromised. The findings therefore challenge normative assumptions embedded in healthcare organisation that patients can and should self‐manage coordination by showing how such expectations may unintentionally disadvantage patients with mental health disorders.

A key conceptual contribution of this study is the identification of what may be termed conditional agency. Participants were formally expected to act as autonomous and coordinating patients, yet their actual capacity to do so was shaped by distress, cognitive overload, fragmented pathways and the degree of relational support available. Agency therefore appeared not as a stable individual trait, but as something produced or constrained through healthcare organisation and professional practice.

### 4.2 Communication as a Clinical and Ethical Practice

The theme of communication as care underscores that communication extends beyond information transfer and constitutes a core clinical practice. Patients described how standardised, fast‐paced consultations and reliance on verbal information undermined their ability to participate, remember and make informed decisions. Predictability, repetition and written information were described as stabilising and anxiety‐reducing.

These findings resonate with research on shared decision‐making and patient‐centred communication, which emphasises the importance of adapting information to patients' capacities and preferences (Coulter and Jenkinson 2005; Shickh et al. 2023). However, our study adds new knowledge by demonstrating how mental health vulnerability fundamentally shapes patients' ability to engage with information. Communication practices that are adequate for the ‘average’ patient may be insufficient or even harmful for patients experiencing anxiety, attention difficulties, or cognitive overload. Thus, communication adaptation should be understood not as an optional accommodation, but as an essential component of equitable care.

### 4.3 Anticipating Mental Health Deterioration During Cancer Treatment

Another important contribution of this study is the demonstration that cancer treatment can actively destabilise mental health, yet such effects are rarely anticipated or integrated into care planning. Patients described sudden escalations in anxiety, panic, insomnia and mood instability, often feeling unprepared and unsupported when symptoms worsened.

While the bidirectional relationship between cancer and mental health has been well documented (Brook and Amaro Calcia 2016; Shalata et al. 2024), our findings show that this knowledge is not consistently operationalised in clinical practice. Patients did not primarily ask for specialised psychiatric interventions, but for acknowledgment, monitoring and shared planning. This highlights a gap between knowledge and practice and underscores the need for anticipatory, integrated approaches that recognise mental health deterioration as a predictable risk rather than an unexpected complication.

### 4.4 Stigma, Epistemic Injustice and Self‐Silencing

The findings also reveal how stigma operates subtly within oncology care, shaping what patients feel able to disclose. These findings also resonate with broader literature on diagnostic overshadowing and inequities in physical healthcare for people living with mental health conditions. Recent work by (Schäfer et al. 2026) highlights that patients frequently describe not being taken seriously, encountering communication barriers and having physical symptoms interpreted primarily through the lens of mental illness. Such processes may delay assessment, weaken trust and reduce opportunities for timely and responsive care. Our findings extend this literature by demonstrating how similar dynamics may also unfold within oncology settings, where highly specialised somatic treatment can coexist with insufficient recognition of psychological vulnerability and relational needs. Patients described hesitating to speak about distress or minimising symptoms due to fears of not being taken seriously. This aligns with research showing that stigma continues to affect healthcare encounters for people with mental illness, even in somatic settings (Phelan et al. 2023; Vass et al. 2025). Conceptually, these findings can be understood through the lens of epistemic injustice, where patients' accounts of their own experiences are implicitly devalued. In this study, stigma was not primarily expressed through overt discrimination, but through silence, lack of inquiry and absence of response. Such dynamics contribute to self‐silencing, which may compromise both emotional support and clinical safety. The study thus contributes new empirical insight into how epistemic injustice unfolds in oncology contexts.

### 4.5 Relational Continuity as a Protective Factor

Despite systemic challenges, patients described relational continuity, being remembered and having trusted companions as key protective factors. These supports were not necessarily formal or resource‐intensive, but relational and low‐threshold. Feeling recognised as a person rather than a diagnosis restored dignity and mitigated fragmentation.

This finding aligns with nursing and psychosocial literature emphasising relational care, continuity and trust (Granek et al. 2019a; Merrick et al. 2022). However, our study extends this work by showing that relational continuity is particularly critical for patients with co‐morbidity, for whom fragmentation and self‐advocacy demands are especially burdensome. Relational anchors functioned as stabilising infrastructures within otherwise fragmented systems.

### 4.6 Contribution to Knowledge and Implications

Taken together, this study contributes new knowledge by illuminating how patients with concurrent cancer and mental health disorders experience oncology care not simply as insufficiently integrated, but as structurally misaligned with their capacities and vulnerabilities. The findings show that patient‐centred care, from the patient perspective, is about recognition, predictability, shared responsibility and relational continuity.

The implications are significant. For clinical practice, the findings suggest that small, relationally attuned adjustments such as adapted communication, explicit acknowledgment of mental health and shared coordination may have a substantial impact. For organisation and policy, the study underscores the need to move beyond siloed models of care and rethink assumptions about patient autonomy and self‐management. Finally, for research, the study highlights the importance of centring patient experiences to understand how complex care is actually lived, while also pointing to the need for further research that explores healthcare professionals' efforts, constraints and clinical reasoning in attempting to meet the complex needs of this population.

From a systems perspective, the findings suggest that vulnerability is amplified when highly specialised services operate through siloed responsibilities, time‐pressured encounters and assumptions of patient self‐management. Conversely, integrated communication pathways, shared responsibility across sectors, continuity of contact persons and psychologically informed oncology practice may function as protective organisational mechanisms. The study therefore points toward a model in which person‐centred cancer care for patients with co‐morbidity depends as much on system design as on individual clinician competence.

### 4.7 Strengths and Limitations

A key strength of this study is its in‐depth qualitative exploration of patient experiences across a diverse group of individuals living with different cancer diagnoses and mental health conditions. The cross‐case analysis enabled the identification of shared patterns and recurring experiences across participants rather than isolated individual accounts. Several limitations should also be acknowledged. First, the study was conducted within a specific healthcare context, which may limit transferability to other settings or healthcare systems. Second, only one male participant was included, meaning that male perspectives may be underrepresented. Third, the study focused exclusively on patients' perspectives and did not include healthcare professionals' views. As a result, important contextual nuances, clinical reasoning and efforts undertaken by professionals may not have been fully captured. Nevertheless, the themes identified resonate with international literature, suggesting that the findings may have broader relevance beyond the immediate study context. The modest sample size, voluntary recruitment and recruitment through clinical settings may also have introduced self‐selection and gatekeeping effects, potentially influencing which experiences were represented.

## 5 Conclusion

Patients living with concurrent cancer and mental health disorders navigate oncology care within systems that strive for person‐centredness, yet are structured around biomedical treatment pathways. This study demonstrates that being met in one's individual needs is less about the absence of care and more about how recognition, adaptation and continuity are enacted in everyday clinical practice—especially at times of heightened vulnerability. Navigating these diagnoses is complex, as cancer treatment requires active engagement while mental health challenges persist in parallel. Supporting this population therefore requires not only clinical competence, but relational attentiveness, anticipatory care and shared responsibility across professional and organisational boundaries. Our findings suggest that diagnostic overshadowing may operate not only through missed diagnosis, but also through reduced recognition of emotional distress, support needs and patients' experiential knowledge.

## Author Contributions

Kim Jørgensen was the principal investigator and main author of the study and manuscript. Maria Estrup, Kathrine Holmstrøm Ougaard, Hanne Elisabeth Bagger Hansen, Kimmie Juul Heine and Sofie Schuster contributed to data collection, interviews and analysis. Karin Piil contributed to study planning, participant recruitment, data analysis and drafting of the manuscript. Helle Pappot contributed with clinical and scientific supervision and critical revision of the manuscript. All authors contributed to the interpretation of the findings, critically revised the manuscript and approved the final version prior to submission.

## Funding

The authors have nothing to report.

## Conflicts of Interest

The authors declare no conflicts of interest.

## Supporting information


**Data S1:** jocn70367‐sup‐0001‐Supinfo.docx.

## Data Availability

The data that support the findings of this study are not publicly available due to the sensitive and potentially identifiable nature of qualitative interview materials and the confidentiality agreements made with participants. In accordance with Wiley's Data Sharing Policy, de‐identified data excerpts or analytic materials (e.g., coding frameworks) may be made available from the corresponding author upon reasonable request and subject to institutional or ethical approval.
