# Supplementary material for: Navigating Concurrent Diagnoses With Cancer and Mental Health Disorder: Patients' Perspectives on Personalised Treatment and Care
Source: J Clin Nurs. 2026 May 21;35(9):3931–41. doi: 10.1111/jocn.70367 (PMC13431843; doi:10.1111/jocn.70367)
Supplement: Supplementary file 1 — Data S1: jocn70367‐sup‐0001‐Supinfo.docx. [file JOCN-35-3931-s001.docx]

Coreq

| **Domain / Item** | **Description for This Study** | **Page No.** |
| --- | --- | --- |
| **Domain 1: Research Team and Reflexivity** |  |  |
| 1. Interviewer/facilitator | One moderator led the focus group; two co-moderators supported. Individual interviews conducted by research team members. | p. 10 |
| 2. Credentials | Interviewers included PhD, MSc, RN-qualified researchers. | p. 10 |
| 3. Occupation | Researchers employed at universities and clinical research departments. | p. 1–2 |
| 4. Gender | Not explicitly described; team includes both genders. | – |
| 5. Experience & training | Team trained in qualitative research; interview guide based on Kvale & Brinkmann and Rogers. | p. 11 |
| 6. Relationship established | No prior therapeutic/supervisory relationship. | p. 10 |
| 7. Participant knowledge of interviewer | Participants knew researchers’ academic roles and study aim. | p. 10 |
| 8. Interviewer characteristics | Roles and motivations disclosed; reflexivity maintained during analysis. | p. 11 |
| **Domain 2: Study Design** |  |  |
| 9. Methodological orientation | Qualitative content analysis (Graneheim & Lundman). | p. 12–13 |
| 10. Sampling | Purposive sampling. | p. 9 |
| 11. Method of approach | Recruitment via managers; voluntary participation. | p. 9 |
| 12. Sample size | 10 in focus group + 2 interviews (n=12). | p. 10 |
| 13. Non-participation | Not reported. | – |
| 14. Setting of data collection | Oncology departments in two Danish hospitals. | p. 8–9 |
| 15. Presence of non-participants | None besides moderators. | p. 10 |
| 16. Description of sample | Oncology nurses, physicians, nurse managers. | p. 8–10 |
| 17. Interview guide | Yes; open-ended; based on Brinkmann & Kvale and Rogers. | p. 11 |
| 18. Repeat interviews | None. | – |
| 19. Audio/visual recording | Audio-recorded. | p. 11 |
| 20. Field notes | Not reported. | – |
| 21. Duration | FG ~60 min; interviews 45–60 min. | p. 10 |
| 22. Data saturation | Not claimed. | – |
| 23. Transcripts returned | No. | – |
| **Domain 3: Analysis and Findings** |  |  |
| 24. Number of data coders | Multiple researchers coded collaboratively. | p. 12 |
| 25. Coding tree description | Meaning units → condensation → codes → subcategories → categories → themes. | p. 13 |
| 26. Derivation of themes | Inductive. | p. 13 |
| 27. Software | NVivo. | p. 12 |
| 28. Participant checking | None. | – |
| 29. Quotations presented | Yes. | p. 14–20 |
| 30. Data consistent with findings | Yes. | p. 14–21 |
| 31. Clarity of major themes | Five major themes described. | p. 14–20 |
| 32. Clarity of minor themes | Minor themes embedded in subcategories. | p. 14–21 |
